# Supplementary material for: Integrating defense and leaf economic spectrum traits in a tropical savanna plant
Source: Front Plant Sci. 2023 Jun 5;14:1185616. doi: 10.3389/fpls.2023.1185616 (PMC10277734; doi:10.3389/fpls.2023.1185616)
Supplement: Supplementary file 1 [file DataSheet_1.docx]

Supplemental information

**Integrating defense and leaf economic spectrum traits in a tropical savanna plant**

Estimation of Herbivory intensity (HI)

Herbivory intensity is defined as the proportion of plant biomass that is consumed by herbivores. To obtain site-specific measures, we estimated HI as 1- ratio of Vegetation Index-based standing biomass and rainfall-based measures of primary productivity.

Primary productivity: We predicted primary productivity across the park using established relationships between rainfall and herbaceous primary productivity. Briefly, we combined data on biomass production and rainfall from several grasslands globally (Gill et al. 2015, Veldhuis et al. 2016), including data from the Serengeti (Sinclair 1975, McNaughton 1985, Ritchie 2014, Veldhuis et al. 2019) and estimated the slope and intercept for the relationship between biomass productivity and growing season rainfall (Fig S1). This yielded the following equation with R^2^ =0.83:

log(Herbaceous productivity) = -0.971 + 1.122*log(growing season rainfall) (1)


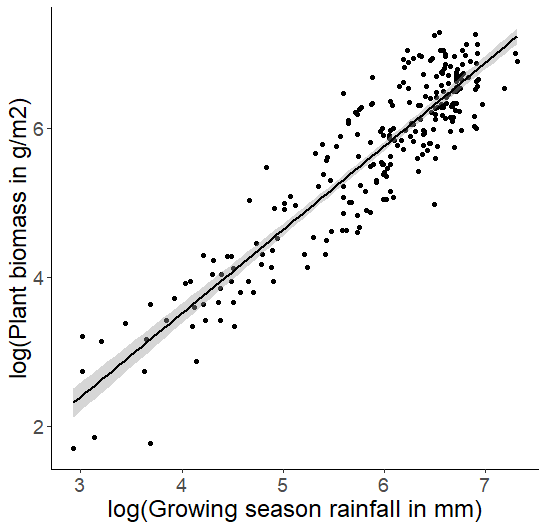


Figure S1: Relationship between rainfall and plant biomass in fenced plots from various sites across the globe (R^2^=0.83).

We then predicted park-wide herbaceous productivity using spatiotemporal rainfall data from the CHIRPS data (Funk et al. 2015). Growing season rainfall estimates were calculated by summing monthly CHIRPS rainfall estimates for the period September-May on a pixel bases with a resolution of 5x5 km using linear models (lm()). Primary productivity was then predicted for each year using equation 1.

Standing biomass: To estimate end of season herbaceous biomass at the park-scale we used Enhanced Vegetation Index (EVI) provided by MODerate-resolution Imaging Spectroradiometer (MODIS) with an approximate 250 x 250 m pixel resolution and 16-day interval between 2009 and 2018 (MOD13Q1) (Didan 2015). We chose the Julian dates (129,145 and 161) as they correspond to the end of the season (Fig S2). We corrected for signals from non-herbaceous evergreen species and soil differences by subtracting the minimum value of EVI (in a year) from all the EVI estimates. To confirm the usefulness of EVI estimates in predicting plant biomass in the Serengeti region, we regressed remotely sensed EVI values against ground-measured biomass data from grazed plots at 8 exclosure sites from 4 non-consecutive years (N=32).


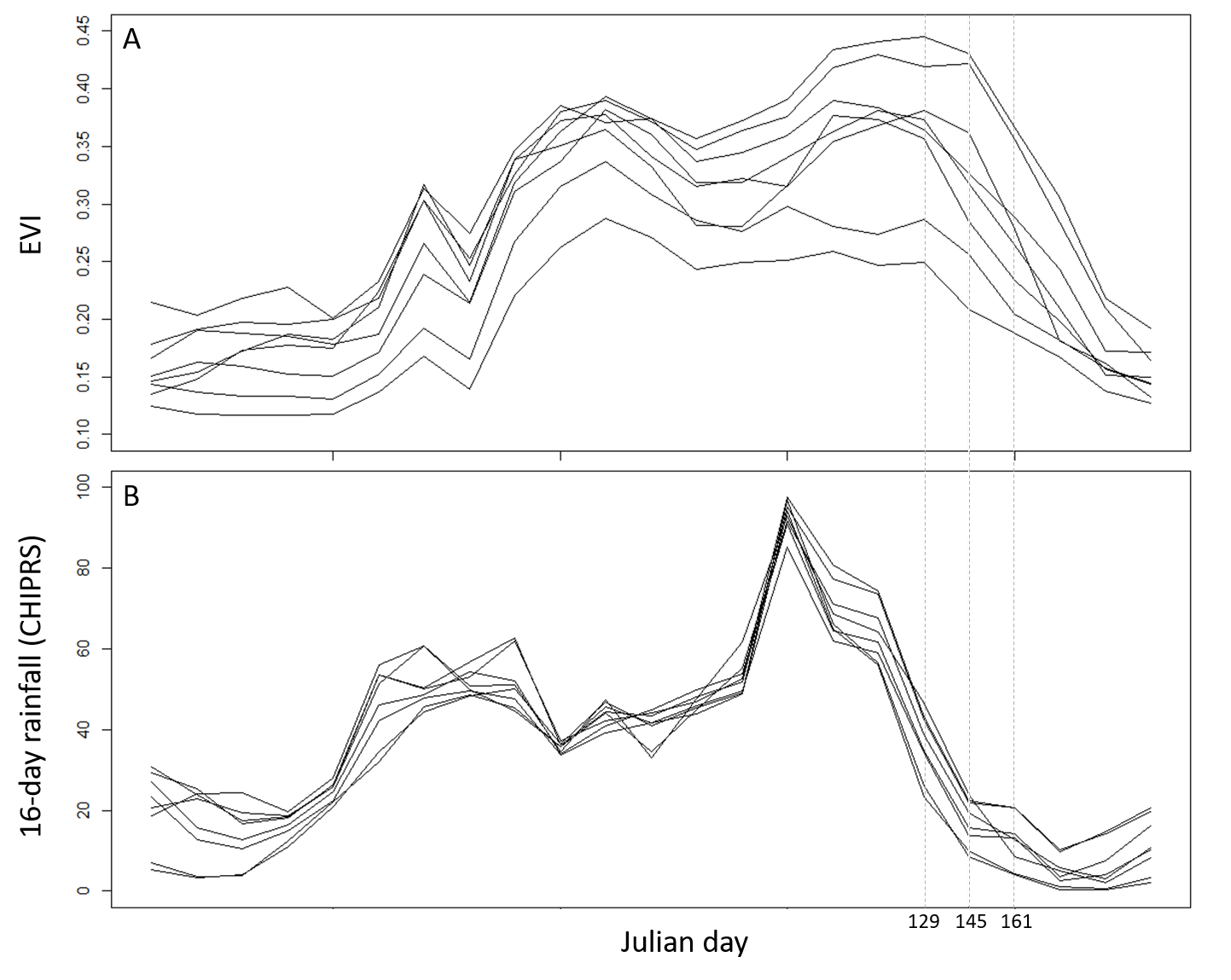


Figure S2: Mean annual variation in EVI (A) and 16-day rainfall (B) for the 8 Long Term Grazing Exclosure sites between 2000-2016. In May (Julian days 129 and 145), rainfall declines marking the beginning of the dry season followed by a drop in EVI and NDVI in June (Julian day 161).

We found that EVI Julian day 145 corrected for minimum EVI proved to best explain the variation in standing biomass (Table S1) and was therefore used for subsequent analyses. End-of-season biomass was then calculated for each year at a resolution of 250x250m at the park-scale using the following equation (2) obtained from the regressions (R^2^=0.45) described above:

Herbaceous biomass = 10^(2.20937+1.14917*EVIcorrected) (2)

Park-wide 10-year average herbivory intensities from 2009-2018 were then calculated using the following equation:

HI = 1 - (Herbaceous biomass/herbaceous productivity) (4)

Table S1: Results from linear regressions between log-transformed plant biomass from the LTGE experiment and corrected (suffix ‘.cor’) or uncorrected Enhanced Vegetation Indices (EVI) for different Julian days (129,145,161).

| Y variable | X variable | Intercept | Estimate (SE) | Mult R2 | Adj R2 | F | df |
| --- | --- | --- | --- | --- | --- | --- | --- |
| log10 (biomass) | EVI.145.cor | 2.209 | 1.149 (0.222) | 0.47 | 0.45 | 26.8 | 1,30 |
| log10 (biomass) | EVI.161.cor | 2.228 | 1.523 (0.318) | 0.44 | 0.42 | 22.8 | 1,28 |
| log10 (biomass) | EVI.129.cor | 2.199 | 1.062 (0.240) | 0.39 | 0.37 | 19.5 | 1,30 |
| log10 (biomass) | EVI.161 | 2.078 | 1.402 (0.304) | 0.43 | 0.41 | 21.2 | 1,28 |
| log10 (biomass) | EVI.145 | 2.093 | 1.096 (0.221) | 0.45 | 0.43 | 24.6 | 1,30 |
| log10 (biomass) | EVI.129 | 2.105 | 0.979 (0.236) | 0.36 | 0.34 | 17.1 | 1,30 |

Note: All associations were statistically significant at alpha=0.05. EVI.145.cor had the highest Adjusted R2 and was therefore used in further analyses.

We then extracted values of HI for each of the 61 sampled sites. All spatial analyses were run in R3.5.1 (R Core Team 2018) using the raster package (Hijmans et al. 2021).

Herbivory Intensity (HI) is essentially an ecosystem-level estimate of the impact of multiple herbivore species on herbaceous biomass and should correlate with herbivore abundance (Staver et al. 2021). As data on herbivore abundance are unavailable for our sites of interest, we tested for associations between HI and herbivore abundance over a narrower region in the central Serengeti, for which multi-year camera trap data are available through the Snapshot Serengeti project (Anderson et al. 2010, Swanson et al. 2015). Briefly, we gathered count data for different herbivore species from the 225 camera traps deployed between 2010 to 2013 and distributed over a 1125km^2^ region. Using the “consensus_data.csv” file, we summed over all browser and mixed-feeding species known to consume *Solanum* species and divided it by the number of days the cameras were deployed, to account for varying search effort among cameras. As there is an inherent scale mismatch between the field of view of the camera and the resolution (250m x 250 m) of the satellite-based herbivory intensity (HI), and errors that inevitably result from estimating animal abundances from aggregated individuals (Burton et al. 2015, Pacifici et al. 2019), we averaged browser abundances over multiple cameras within 5 x 5 km grid cells. To match scales of herbivore abundance and HI, we averaged the HI estimates within 5 x 5 km grids. We then compared the average herbivore abundance with satellite-based estimates of HI for *N* = 58 grid cells. We repeated the analysis for predominantly grazing species (wildebeest, zebra, buffalo, topi, hartebeest, and gazelles) to test if HI was better associated with risk to graminoids. Given the scarcity of data on large herbivore distribution at the landscape scale, we argue that EVI- based estimate of herbivory intensity provides a proxy for risk from herbivory at different sites.

Results:

Herbivory intensity varied from 0.25 to 0.53, implying that 25 to 53% of the aboveground biomass was consumed by herbivores across the 43 sites at which *S. incanum* was present. Although the regions with camera traps spanned a narrower range of HI from 0.36 to 0.44, HI was positively associated with browser and mixed-feeding herbivore abundances (slope (SE): linear term: 0.012 (0.004), p=0.001; insignificant quadratic term: -0.003 (0.002), p=0.08, R^2^=0.15) (Fig 1a), but not for grazer and mixed-feeding herbivore abundances (0.006 (0.003), p=0.06, R^2^=0.04) (Fig 1b).


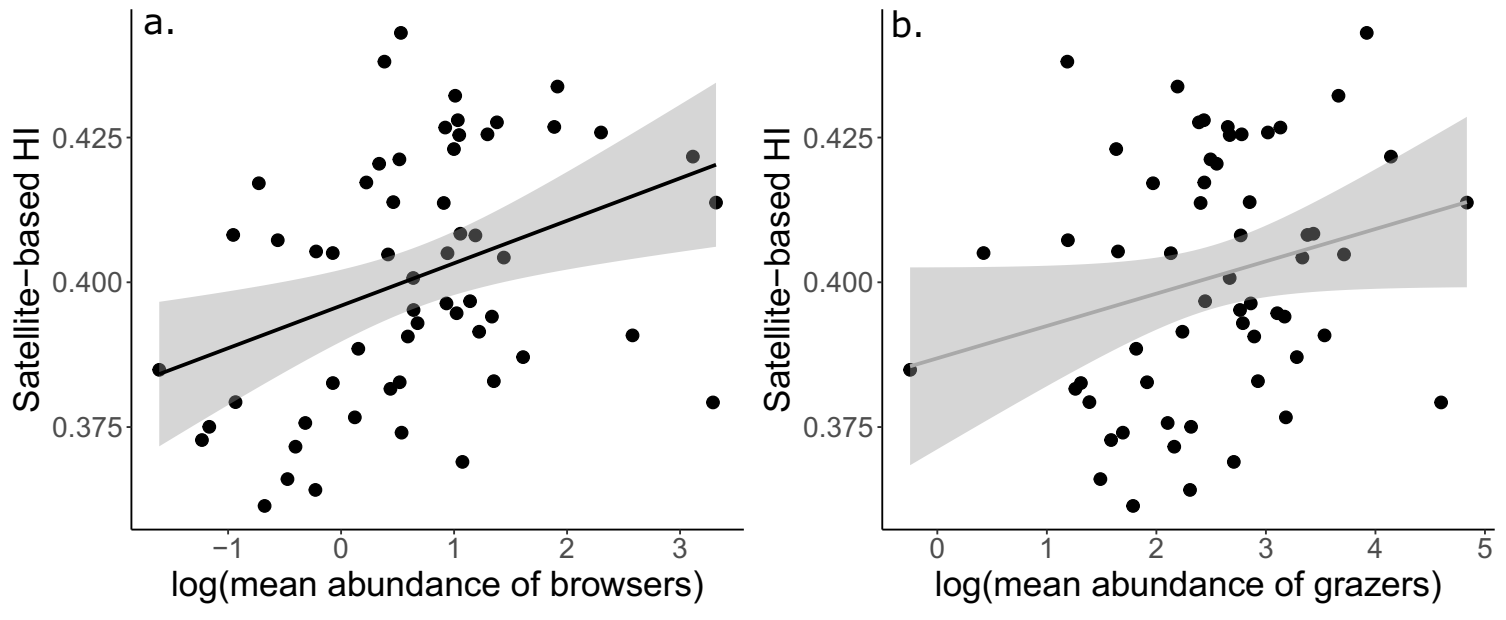


Figure 1: Association between satellite-based herbivory intensity (HI) and log-transformed a) browser abundance (species that consume *Solanum*), and b) grazer and mixed-feeding species abundance based on the Snapshot Serengeti camera trap data. The dots represent abundance and HI estimates in 5 x 5 km in the central Serengeti, dark and grey lines represent significant and insignificant relationships, respectively, and grey areas represent the 95% confidence intervals.

Effects of large mammalian herbivores on plant defenses have been harder to address at the landscape scale due to the difficulties associated with measuring risk from herbivores. Although we were able to show that herbivory intensity was positively associated with browser relative abundance, the relationship was weak, as might be expected. Camera traps often feature high sampling variance for mobile, highly aggregated animals, and detection probabilities may vary with species identity, herd size or animal behavior (Treves et al 2010, Burton et al 2015, Kolowski and Forrester 2017). Moreover, the central Serengeti where the camera traps are placed is a region with relatively high herbivore activity and had a narrow range of HI (0.36 to 0.45) as compared to the range in the rest of our study (0.25 to 0.55). This coupled with the fact that estimation of HI contains errors from the relationship between rainfall and ungrazed biomass driven in part by litter accumulation may also have limited our ability to find a stronger correlation. Yet, we detected statistically significant associations between HI and browser abundance, and between HI and spine density in Solanum, which suggests that HI is an important axis of variation for plant defenses, likely due to its correlation with browser abundances. Additionally, the lack of association between HI and grazer abundance also suggests that HI is a reliable measure of risk from browsing rather than grazing species, likely because EVI-based estimates of plant biomass may be disproportionately affected by herbaceous vegetation which tends to be greener than the surrounding grasses. Thus, vegetation index-based HI offered us a chance to explore patterns in defense traits along a gradient of risk from herbivores that was independent of the plant defense levels, and provides an alternative method for estimating herbivory intensity.

References:

Anderson, T. M., J. G. C. Hopcraft, S. Eby, M. Ritchie, B. James, H. Olff, T. Michael, C. Hopcraft, and B. Grace. 2010. Landscape-scale analyses suggest both nutrient and antipredator advantages to Serengeti herbivore hotspots. Ecology 91:1519–1529.

Burton, A. C., E. Neilson, D. Moreira, A. Ladle, R. Steenweg, J. T. Fisher, E. Bayne, and S. Boutin. 2015. REVIEW: Wildlife camera trapping: a review and recommendations for linking surveys to ecological processes. Journal of Applied Ecology 52:675–685.

Didan, K. 2015. MOD13Q1 MODIS/Terra vegetation indices 16-day L3 global 250m SIN grid V006. NASA EOSDIS Land Processes DAAC 10.

Funk, C., P. Peterson, M. Landsfeld, D. Pedreros, J. Verdin, S. Shukla, G. Husak, J. Rowland, L. Harrison, A. Hoell, and J. Michaelsen. 2015. The climate hazards infrared precipitation with stations—a new environmental record for monitoring extremes. Scientific Data 2:150066.

Gill, R. A., R. H. Kelly, W. J. Parton, K. A. Day, R. B. Jackson, J. A. Morgan, J. M. O. Scurlock, L. L. Tieszen, J. R. Vande Castle, D. S. Ojima, and X. Zhang. 2015. NPP Grassland: Consistent Worldwide Site Estimates, 1954-1990, R1. ORNL DAAC.

Hijmans, R. J., J. van Etten, M. Sumner, J. Cheng, D. Baston, A. Bevan, R. Bivand, L. Busetto, M. Canty, B. Fasoli, D. Forrest, A. Ghosh, D. Golicher, J. Gray, J. A. Greenberg, P. Hiemstra, K. Hingee, I. for M. A. Geosciences, C. Karney, M. Mattiuzzi, S. Mosher, B. Naimi, J. Nowosad, E. Pebesma, O. P. Lamigueiro, E. B. Racine, B. Rowlingson, A. Shortridge, B. Venables, and R. Wueest. 2021. raster: Geographic Data Analysis and Modeling.

McNaughton, S. J. 1985. Ecology of a Grazing Ecosystem : The Serengeti. Ecological Monographs 55:259–294.

Pacifici, K., B. J. Reich, D. A. W. Miller, and B. S. Pease. 2019. Resolving misaligned spatial data with integrated species distribution models. Ecology 100:e02709.

R Core Team. 2018. R: The R Project for Statistical Computing. https://www.r-project.org/.

Ritchie, M. E. 2014. Plant compensation to grazing and soil carbon dynamics in a tropical grassland. PeerJ 2:e233.

Sinclair, A. R. E. 1975. The Resource Limitation of Trophic Levels in Tropical Grassland Ecosystems. Journal of Animal Ecology 44:497–520.

Staver, A. C., J. O. Abraham, G. P. Hempson, A. T. Karp, and J. T. Faith. 2021. The past, present, and future of herbivore impacts on savanna vegetation. Journal of Ecology 109:2804–2822.

Swanson, A., M. Kosmala, C. Lintott, R. Simpson, A. Smith, and C. Packer. 2015. Snapshot Serengeti, high-frequency annotated camera trap images of 40 mammalian species in an African savanna. Scientific Data 2:150026.

Veldhuis, M. P., H. F. Fakkert, M. P. Berg, and H. Olff. 2016. Grassland structural heterogeneity in a savanna is driven more by productivity differences than by consumption differences between lawn and bunch grasses. Oecologia 182:841–853.

Veldhuis, M. P., M. E. Ritchie, J. O. Ogutu, T. A. Morrison, C. M. Beale, A. B. Estes, W. Mwakilema, G. O. Ojwang, C. L. Parr, J. Probert, P. W. Wargute, J. G. C. Hopcraft, and H. Olff. 2019. Cross-boundary human impacts compromise the Serengeti-Mara ecosystem. Science 363:1424–1428.
